# Videomicroscopy as a tool for investigation of the microcirculation in the newborn

**DOI:** 10.14814/phy2.12941

**Published:** 2016-09-30

**Authors:** Ian M. R. Wright, Joanna L. Latter, Rebecca M. Dyson, Chris R. Levi, Vicki L. Clifton

**Affiliations:** ^1^ Hunter Medical Research InstituteNewcastleNew South WalesAustralia; ^2^ Discipline of Paediatrics and Child HealthSchool of Medicine and Public HealthUniversity of NewcastleNewcastleNew South WalesAustralia; ^3^ Kaleidoscope Neonatal Intensive Care UnitJohn Hunter Children's HospitalNewcastleNew South WalesAustralia; ^4^ Graduate School of Medicine and Illawarra Health and Medical Research InstituteUniversity of WollongongWollongongNew South WalesAustralia; ^5^ Mater Medical Research InstituteUniversity of QueenslandBrisbaneQueenslandAustralia

**Keywords:** Capillary, cardiovascular, microcirculation, newborn, videomicroscopy

## Abstract

The perinatal period remains a time of significant risk of death or disability. Increasing evidence suggests that this depends on microcirculatory behavior. Sidestream dark‐field orthogonal polarized light videomicroscopy (OPS) has emerged as a useful assessment of adult microcirculation but the values derived are not delineated for the newborn. We aimed to define these parameters in well term newborn infants. Demographic details were collected prospectively on 42 healthy term neonates (*n* = 20 females, *n* = 22 males). OPS videomicroscopy (Microscan) was used to view ear conch skin microcirculation at 6, 24, and 72 h of age. Stored video was analyzed by a masked observer using proprietary software. There were no significant differences between the sexes for any structural parameters at any time point. There was a significant increase over time in small vessel perfusion in female infants only (*P* = 0.009). A number of 6‐ and 72‐h measurements were significantly correlated, but differed from the 24‐h values. These observations confirm the utility of the ear conch for neonatal microvascular videomicroscopy. They provide a baseline for studies into the use of OPS videomicroscopy in infants. The changes observed are comparable with previous studies of term infants using these and other microvascular techniques. It is recommended that studies for examining the mature neonatal microvascular structure be delayed until 72 h of life, but studies of the physiology of cardiovascular transition should include the 24‐h time point after delivery.

## Introduction

The perinatal period remains a time of significant risk of death or disability. In the developed world, it is the major contributor to infant mortality with 10% of infants likely to die when born too early (Parry et al. 2003). In the developing world, this is even more pronounced (Ngoc et al. 2006). In addition, illness around this time is associated with significant long‐term morbidity with the associated increase in health care costs (Doyle 2004).

Most infants who die become sick in the first 72 h of life and understanding this period of rapid cardiovascular transition is crucial to modifying outcomes. There is increasing evidence that the physiology and pathophysiology in this period is dependent on the behavior of the microcirculation. In preterm infants, increased microvascular flow at 24 h of age has been closely associated with poor outcomes including both death and illness severity (Wright et al. 2012). In addition, it is known that poor outcomes in the perinatal period are strongly associated with male sex (Kent et al. 2012); there is now increasing evidence that the dysregulation of the microcirculation, associated with poor outcomes, are more evident in male infants (Stark et al. 2008b; Dyson et al. 2014a).

The majority of recent studies in newborn microcirculation have utilized laser Doppler flowmetry as a noninvasive tool, suitable for use in the human newborn. This technique, however, provides no information of the circulatory structure underlying the observations of microvascular function. While it is highly likely that the described differences are due to functional changes, they may also be due to significant structural changes related to angiogenesis, apoptosis (Pladys et al. 2005; Duval et al. 2007), or differential perfusion of different components of the microcirculation. A potential way of clarifying some of these structural questions is direct noninvasive visualization of the microcirculation in vivo using sidestream dark‐field orthogonal polarized light videomicroscopy (OPS). OPS has emerged as a potentially useful tool for assessment of the microcirculation in adults (Vincent and De Backer 2013; Abdo et al. 2014; Donndorf et al. 2014), but the parameters derived have not been fully delineated for the newborn age group.

The aim of this study was to provide OPS data for all parameters obtained using a commonly used analysis package (AVA v3.0; MicroVision Medical, Amsterdam, The Netherlands) in the healthy term newborn infant. In order to understand the physiology and pathophysiology of the microcirculation in the perinatal period in sicker term infant, or in the preterm infant, it is first necessary to define these values in this well control population. In view of previous study findings using laser Doppler, a priori analyses of the effects of postnatal age and of sex were undertaken.

## Materials and Methods

Expectant mothers were recruited into the “Cardiovascular Adaptation of the Newborn Study 2 (2CANS)” after informed consent following presentation to the antenatal clinic of the John Hunter Hospital, Newcastle, Australia and included if their pregnancy proceeded to term. This study was a planned substudy of a larger study, stratified by gestational age at delivery (very preterm: ≤28 weeks, preterm 29–36 weeks, term ≥37 weeks), based on the protocol of our previous study (CANS1) (Stark et al. 2008a,b). A diagnosis of hypoxic ischemic encephalopathy, congenital malformations, chromosomal disorders, or known congenital infection excluded admission to this study. All study protocols were approved by the ethics committees at the John Hunter Hospital (Hunter New England Local Health District) and the University of Newcastle.

Demographic data were obtained prospectively from the medical record of mother or baby as required, both for baseline data and status at each of the time points. The infants were examined at 6 (±1), 24 (±2), and 72 (±6) h of age. The study time points were based upon our previous observations, in term and preterm infants, of microcirculatory flow changes using laser Doppler flowmetry (Stark et al. 2008a,b; Dyson et al. 2014a). Study timing also depended upon the availability of the infant, operator, and equipment.

**Figure 1:**
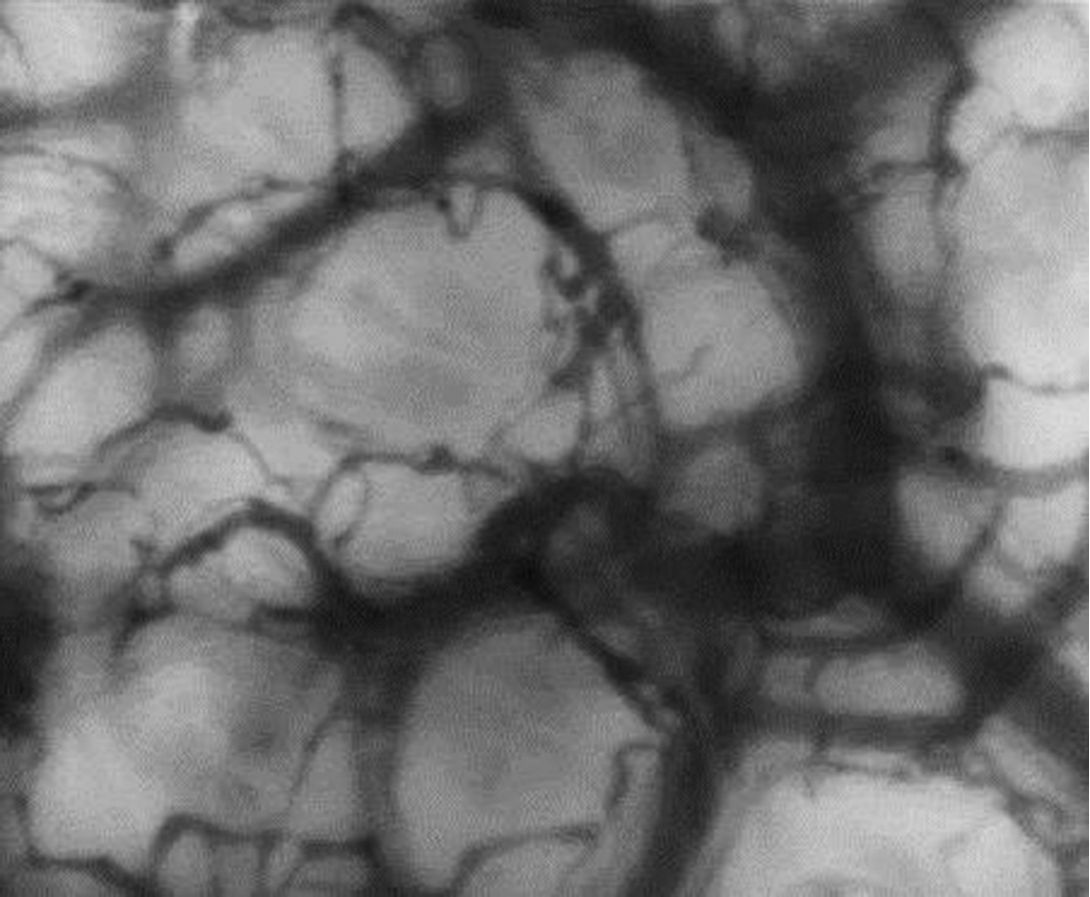
Stacked frames (*n* = 100) from a single video of OPS videomicroscopy from ear conch of a term female infant on day 1 of life.

The sidestream dark‐field orthogonal polarized light videomicroscope camera (Microscan; MicroVision Medical) was used to acquire images. This methodology is described in full elsewhere (De Backer et al. 2007; Trzeciak et al. 2007) but, in brief, a polarized light shone from the end of the instrument into the tissues, above which it was held, barely in contact with a thin surface film of white soft paraffin. The standard single use 5× disposable lens was used (MicroVision Medical). The image was taken on the ear conch, using the nondependent ear. Previous studies have used a variety of body sites for OPS recording, but we chose the ear conch for a number of reasons: it is derived from the same carotid circulation as the widely reported, and clinically significant, sublingual circulation used in adult studies (Vincent and De Backer 2009, 2013); it is physically easy to undertake the study (resting the hand lightly on the side of the infants head or the bed adjacent to the head); it does not involve unwrapping the infant, which is important for thermoregulatory stability and facilitates the infant settling; finally, it only requires one operator, as there is no requirement to hold the arm out of the way (Schwepcke et al. 2013) nor adjust for breathing movement. Others have recently compared this site to previous neonatal methodologies (Alba‐Alejandre et al. 2013). Polarized light traveled into the tissue where it underwent decoherence. The reflected decoherent light passed through the receiving polarized filter to the CCD of the video camera to give, in effect, a deep light source behind the vessels of the microvasculature. The wavelength of light used was 530 nm (green) and so the blood columns within the vessels appeared black (Fig. 1). The image resolution of the system is 720 × 480 pixels with 1.45 *μ*m/pixel spatial resolution, a field of view of 1044 × 758 *μ*m, and 30 frames per second temporal resolution. A duration of 10 sec of recording was attempted for each occasion and this was repeated for a total of three to five recordings, as able. Following analog to digital conversion, the pictures were stored until data analysis was undertaken using proprietary software (AVA) and according to published consensus approaches for this technique (De Backer et al. 2007; Bezemer et al. 2011; Lehmann et al. 2014). An observer, masked to the clinical details, adjusted the image quality for maximum clarity, as per AVA software instructions. Hundred (±30) images were stacked for the structural assessments. Images of poor quality were rejected from further analysis. Semiautomated vessel identification, delineation, and flow attribution were undertaken and then specific flow velocities were established for each vessel type (small: 1–25 *μ*m, medium: 25–50 *μ*m, and large 50–100 *μ*m, predefined defaults within AVA) using time velocity diagrams with 3–6 white cell tracks within the red cell column averaged for each vessel velocity. Between 1 and 5, recordings (median 3) were made for each session on each infant and these were then averaged to give the final values used for each infant at each time point, for use in subsequent statistical analyses.

Statistical analysis was performed for all infants and also for male and female infants separately, as decided a priori. Data were analyzed using SPSS Statistics v20 (IBM Corp., New York, NY). Most data were normally distributed, but if the group numbers were low, appropriate nonparametric testing was used to confirm significance. In view of the known sex differences in microcirculation previously reported (Stark et al. 2008b), correlations were undertaken using partial correlation testing with adjustment for infant sex.

For ease of reporting the parameter values described for these well term infants are reported by grouping parameters into separate tables as follows.

### Structural (quantitative) indices

Detected vessel length, vessel density, vessel surface area percentage, De Backer score, small vessel length, small vessel length percentage, small vessel perfused length, small vessel perfused length percentage, medium vessel length, medium vessel length percentage, medium vessel perfused length, medium vessel perfused length percentage, large vessel length, large vessel length percentage, large vessel perfused length, large vessel perfused length percentage.

### Functional indices (semiquantitative)

Sluggish flow small vessels, continuous flow small vessels, sluggish flow medium vessels, continuous flow medium vessels, sluggish flow large vessels, continuous flow large vessels, small vessel total vessel density (TVD), small vessel perfused vessel density (PVD), small vessel proportion perfused vessels (PPV), small vessel microvascular flow index (MFI), other vessel TVD, other vessel PVD, other vessel PPV, other vessel MFI, all vessel TVD, all vessel PVD, all vessel PPV, all vessel MFI.

### Velocity/flow indices (quantitative)

Small vessel average velocity, medium vessel average velocity, large vessel average velocity, all vessel average velocity, small vessel average flow, medium vessel average flow, large vessel average flow, all vessel average flow.

Unless otherwise stated, values are for a stacked field of view obtained within the program (AVA), as in Figure 1. All tabled results are to two significant figures unless otherwise stated.

## Results

Forty‐two term singleton babies were recruited to this study (Table 1). All were normothermic; one female infant was managed briefly on the neonatal unit with continuous positive airway pressure with an eventual diagnosis of transient tachypnea of the newborn. No baby required resuscitation, with mean Apgar scores of 9 at 1 min (range: 6–9), 9 at 5 min (range: 9–10), and 10 at 10 min (range: 9–10). Four (9.5%) infants were small for gestational age (SGA, <10th centile) but none <3rd centile. No baby was delivered following evidence of significant antepartum hemorrhage or chorioamnionitis. There were three mothers who smoked at the beginning of pregnancy, all with male babies: one a moderate smoker (20 cigs/day) and two light smokers (1–5 cigs/day). All continued to smoke at some level throughout pregnancy. No infants were exposed to indomethacin or magnesium treatment perinatally.

**Table 1:** Table of demographics

	Total (*N* = 42)		Females (*N* = 20)		Males (*N* = 22)		*P* b F versus M
	Mean	95% CI	Mean	95% CI	Mean	95% CI	
Gestational age (completed weeks)	39	38–41	39	38–41	40	38–41	NS^a^
Birth weight (g)	3520	2880–4240	3416	2803–4103	3614	2880–4250	NS
Birth weight percentile	60	8–97	57	6–92	63	8–98	NS
SGA, *n* (%)	4 (9.5%)		2 (10%)		2 (9.1%)		NS
Head circumference (cm)	35	33–38	35	32–37	36	34–38	0.002
Head circumference percentile	57	14–97	48	6–89	65	26–97	0.03
Ratio HC%:Bwt%	1.5	0.3–3.3	1.1	0.2–3.1	1.9	0.4–4.7	NS
Maternal BMI (kg/m^2^)	26.4	19.1–36.3	25.9	19.6–37.4	26.8	19.0–36.3	NS
Placental weight (g)	688	370–1010	673	320–1010	701	435–1070	NS
Placental wt:Bwt ratio	0.19	0.1–0.25	0.2	0.1–0.25	0.19	0.13–0.25	NS
Smokers, *n* (%)	3 (7%)		0 (0%)		3 (14%)		NS
Cesarean section – elective, *n* (%)	13 (31%)		7 (35%)		6 (27%)		NS
Cesarean section – total, *n* (%)	19 (45%)		9 (45%)		10 (45%)		NS
Maternal vasoactive medication, *n* (%)	7 (17%)		5 (25%)		2 (9%)		NS

a Calculated on gestation in days.

b Two‐sample *t*‐test for continuous variables; Chi‐squared or Fishers exact for proportions.

There were 20 (48%) female infants, 10% SGA; *n* = 9 delivered by cesarean section, *n* = 3 by assisted vaginal delivery, and *n* = 8 by normal vaginal delivery. Apgar scores were 9 at 1 min (range: 6–9), 9 at 5 min (range: 9–10), and 10 at 10 min (range: 9–10). One mother had a history of gestational diabetes, five of gestational hypertension (very mild), and one a renal condition (recurrent urinary tract infections; normal renal scan and function). One mother of a female infant had prolonged rupture of membranes (>24 h), one mother of a female infant received nifedipine, and four received beta‐blockers.

There were 22 (52%) male infants, 9.1% SGA; *n* = 10 delivered by cesarean section, *n* = 4 by assisted vaginal delivery, and *n* = 8 by normal vaginal delivery. Apgar scores were 9 at 1 min (range: 6–9), 9 at 5 min (range: 9–10), and 9 at 10 min (range: 9–10). Four mothers of male infants had gestational hypertension (very mild) and one a renal condition (recurrent urinary tract infections; normal renal scan and function). Two mothers of male infants had prolonged rupture of membranes (>24 h), one of which received a course of steroids greater than 1 week prior to delivery. No male infants were exposed to nifedipine perinatally, but two mothers did receive treatment with beta‐blockers.

Due to investigator, equipment, and infant availability, not all infants were seen at all three time points. Where a scan was attempted, pictures were recorded in 96% of occasions. The percentage of successful image acquisition was 98% at 6 h, 94% at 24 h, and 87% at 72 h. The rate of high‐quality images suitable for analysis from these was thus 88%, with others rejected for a variety of reasons (sequence too short, image clarity, or evidence of pressure effects; Trzeciak et al. 2007). Overall, there were 37 at 6 h, 39 at 24 h, and 18 at 72 h after quality control measures. Five infants had one recording, 22 infants two recordings, and 15 infants had recordings at all three time points.

While most parameters were normally distributed over the larger group, we have reported both means and medians to give results that will allow comparisons with future studies. These are displayed in functional parameter groups, as described above, and by age of life in Tables 2, 3, 4. For all individual parameters and all time points, there were no differences between the sexes and so these have been amalgamated to produce these tables of OPS videomicroscopy values in well newborn infants.

**Table 2:** Videomicroscopy measures at 6 h of age in the term newborn: (a) structural; (B) functional and (C) velocity/flow

Videomicroscopy parameter	Mean	SD	Lower 95%	Upper 95%	Median	25th Quartile	75th Quartile
(A) Structural							
Detected vessel length (mm)	7.0	1.2	4.8	9.1	6.9	6.1	7.6
Vessel density (mm/mm^2^)	18	3.1	13	24	18	16	20
Vessel surface area percentage	29	3.5	22	34	29	26	31
De Backer score (1/mm)	12	2.6	8.3	17	12	11	13
Small vessel length (mm)	5.8	1.3	3.1	8.1	5.8	5.0	6.4
Small vessel length percentage	82	7.1	70	93	82	79	88
Small vessel perfused length (mm)	5.8	1.4	3.1	8.1	5.8	5.0	6.4
Small vessel perfused length percentage	82	7.6	69	93	81	79	89
Medium vessel length (mm)	1.1	0.35	0.52	1.8	1.1	0.94	1.4
Medium vessel length percentage	17	5.9	7.0	27	17	12	20
Medium vessel perfused length (mm)	1.1	0.36	0.52	1.8	1.1	0.87	1.5
Medium vessel perfused length percentage	17	6.2	7.0	27	18	11	21
Large vessel length (mm)	0.11	0.17	0.01	0.22	0.05	0.02	0.15
Large vessel length percentage	1.8	3.4	0.05	3.8	0.59	0.19	2.2
Large vessel perfused length (mm)	0.13	0.19	0.01	0.56	0.05	0.017	0.15
Large vessel perfused length percentage	2.1	3.7	0.06	9.4	0.59	0.21	2.2
(B) Functional							
Small vessels sluggish flow (%)	3.6	5.2	0.0	16	1.1	0.0	5.2
Small vessels continuous flow (%)	74	20	0.0	92	78	71	84
Medium vessels sluggish flow (%)	0.24	0.90	0.0	3.2	0.0	0.0	0.0
Medium vessels continuous flow (%)	16	7.2	0.0	29	16	10	20
Large vessels sluggish flow (%)	a	a	a	a	a	a	a
Large vessels continuous flow (%)	1.2	3.1	0.0	9.9	0.01	0.0	0.93
Small vessels TVD (mm/mm^2^)	15	3.6	8.5	22	15	13	18
Small vessels PVD (mm/mm^2^)	15	3.7	8.5	22	15	13	18
Small vessels PPV (%)	82	7.8	69	93	82	78	89
Small vessels MFI	2.9	0.14	2.6	3.0	3.0	3.0	3.0
Other vessels TVD (mm/mm^2^)	3.1	0.93	1.5	4.8	3.1	2.4	3.8
Other vessels PVD (mm/mm^2^)	3.2	1.0	1.5	4.8	3.2	2.3	3.9
Other vessels PPV (%)	18	7.8	6.6	31	18	11	22
Other vessels MFI	3.0	0.12	2.6	3.0	3.0	3.0	3.0
All vessels TVD (mm/mm^2^)	18	3.1	13	24	18	16	20
All vessels PVD (mm/mm^2^)	18	3.1	13	24	18	16	20
All vessels PPV (%)	100	0	100	100	100	100	100
All vessels MFI	3.0	0.13	2.6	3.0	3.0	3.0	3.0
(C) Velocity/flow							
Small vessel average velocity (*μ*m/sec)	390	135	170	730	380	310	440
Medium vessel average velocity (*μ*m/sec)	400	160	190	680	390	290	530
Large vessel average velocity (*μ*m/sec)	a	a	a	a	a	a	a
All vessel average velocity (*μ*m/sec)	404	135	238	727	389	328	465
Small vessel average flow (*μ*L/sec)	79 000	36 000	31 000	131 000	75 000	52 000	98 000
Medium vessel average flow (*μ*L/sec)	34 0000	190 000	120 000	690 000	290 000	220 000	420 000
Large vessel average flow (*μ*L/sec)	a	a	a	a	a	a	a
All vessel average flow (*μ*L/sec)	150 000	98 000	39 000	390 000	110 000	76 000	190 000

a Unable to calculate due to small numbers.

**Table 3:** Videomicroscopy measures at 24 h of age in the term newborn: (A) structural; (B) functional; and (C) velocity/flow

Videomicroscopy parameter	Mean	SD	Lower 95%	Upper 95%	Median	25th Quartile	75th Quartile
(A) Structural							
Detected vessel length (mm)	6.9	1.1	5.0	8.7	6.9	6.0	7.7
Vessel density (mm/mm^2^)	18	3	13	22	18	15	20
Vessel surface area percentage	29	4	22	36	29	26	31
De Backer score (1/mm)	12	2.5	7.3	16	12	10	14
Small vessel length (mm)	5.6	1.3	3.2	8.0	5.5	4.7	6.6
Small vessel length percentage	81	9	63	92	83	75	89
Small vessel perfused length (mm)	5.8	1.2	4.0	8.0	5.6	4.9	6.6
Small vessel perfused length percentage	82	8	66	92	82	75	89
Medium vessel length (mm)	1.1	0.5	0.4	2.1	1.0	0.8	1.4
Medium vessel length percentage	17	8	6	34	15	11	22
Medium vessel perfused length (mm)	1.1	0.5	0.4	1.9	1.0	0.8	1.4
Medium vessel perfused length percentage	16	8	5	28	14	10	21
Large vessel length (mm)	0.23	0.32	0.01	0.82	0.09	0.02	0.33
Large vessel length percentage	3.5	5.8	0.1	16	1.1	0.2	4.0
Large vessel perfused length (mm)	0.28	0.38	0.01	1.3	0.06	0.02	0.40
Large vessel perfused length percentage	4.2	6.9	0	24	0.6	0.2	4.7
(B) Functional							
Small vessels sluggish flow (%)	2.9	4.6	0.0	14	0.0	0.0	4.2
Small vessels continuous flow (%)	79	9.5	62	93	77	73	88
Medium vessels sluggish flow (%)	0.6	1.5	0.0	4.3	0.0	0.0	0.0
Medium vessels continuous flow (%)	15	8.1	4.2	27	13	9.1	23
Large vessels sluggish flow (%)	0.0	0.0	0.0	0.0	0.0	0.0	0.0
Large vessels continuous flow (%)	2.3	5.5	0.0	16	0.0	0.0	0.7
Small vessels TVD (mm/mm^2^)	15	3.4	7.9	20	15	12	18
Small vessels PVD (mm/mm^2^)	15	3.3	9.8	20	15	13	18
Small vessels PPV (%)	82	8.6	66	93	83	75	90
Small vessels MFI	2.9	0.1	2.8	3.0	3.0	3.0	3.0
Other vessels TVD (mm/mm^2^)	3.2	1.1	1.5	5.1	3.3	2.2	4.0
Other vessels PVD (mm/mm^2^)	3.1	1.2	1.5	4.8	3.1	2.3	3.9
Other vessels PPV (%)	18	8.7	6.6	34	17	10	25
Other vessels MFI	3.0	0.1	2.7	3.0	3.0	3.0	3.0
All vessels TVD (mm/mm^2^)	18	2.8	13	22	19	16	20
All vessels PVD (mm/mm^2^)	18	2.5	13	22	19	16	20
All vessels PPV (%)	100	0.2	100	100	100	100	100
All vessels MFI	3.0	0.1	2.8	3.0	3.0	3.0	3.0
(C) Velocity/flow							
Small vessel average velocity (*μ*m/sec)	430	110	270	640	400	370	490
Medium vessel average velocity (*μ*m/sec)	420	140	190	740	420	340	510
Large vessel average velocity (*μ*m/sec)	a	a	a	a	a	a	a
All vessel average velocity (*μ*m/sec)	440	110	260	640	430	370	510
Small vessel average flow (*μ*L/sec)	79 000	29 000	35 000	130 000	83 000	56 000	98 000
Medium vessel average flow (*μ*L/sec)	340 000	160 000	130 000	770 000	340 000	210 000	400 000
Large vessel average flow (*μ*L/sec)	a	a	a	a	a	a	a
All vessel average flow (*μ*L/sec)	130 000	94 000	43 000	290 000	100 000	73 000	170 000

a Unable to calculate due to small numbers.

**Table 4:** Videomicroscopy measures at 72 h of age in the term newborn: (A) structural; (B) functional; and (C) velocity/flow

Videomicroscopy parameter	Mean	SD	Lower 95%	Upper 95%	Median	25th Quartile	75th Quartile
(A) Structural							
Detected vessel length (mm)	7.2	1.3	4.2	8.6	7.6	6.9	8.1
Vessel density (mm/mm^2^)	18	3.2	10	22	19	17	21
Vessel surface area percentage	29	3.0	25	34	29	26	32
De Backer score (1/mm)	12	2.1	5.9	16	13	11	14
Small vessel length (mm)	5.9	1.4	2.6	7.8	5.7	5.5	6.9
Small vessel length percentage	81	7.5	62	92	80	76	86
Small vessel perfused length (mm)	6.3	1.1	4.2	7.8	6.6	5.5	7.2
Small vessel perfused length percentage	83	6.3	74	92	82	78	89
Medium vessel length (mm)	1.2	0.39	0.67	1.8	1.2	0.91	1.6
Medium vessel length percentage	18	7.3	8.4	36	18	11	23
Medium vessel perfused length (mm)	1.2	0.40	0.67	1.8	1.1	0.88	1.6
Medium vessel perfused length percentage	16	5.9	8.4	24	16	11	22
Large vessel length (mm)	0.094	0.11	0.01	0.39	0.065	0.01	0.11
Large vessel length percentage	1.4	1.6	0.07	4.9	1.0	0.15	1.6
Large vessel perfused length (mm)	0.11	0.12	0.01	0.39	0.09	0.01	0.13
Large vessel perfused length percentage	1.5	1.8	0.07	4.9	1.2	0.15	1.9
(B) Functional							
Small vessels sluggish flow (%)	2.5	3.8	0.0	11	1.1	0.0	2.4
Small vessels continuous flow (%)	80	5.3	72	90	80	77	84
Medium vessels sluggish flow (%)	0.3	1.1	0.0	4.0	0.0	0.0	0.0
Medium vessels continuous flow (%)	16	6.2	7.9	25	14	11	23
Large vessels sluggish flow (%)	0.0	0.0	0.0	0.0	0.0	0.0	0.0
Large vessels continuous flow (%)	0.9	1.7	0.0	4.9	0.1	0.0	1.0
Small vessels TVD (mm/mm^2^)	15	3	6	20	14	13	17
Small vessels PVD (mm/mm^2^)	15	4	7	20	16	14	18
Small vessels PPV (%)	80	13	42	95	81	77	86
Small vessels MFI	3	0.2	2	3	3	3	3
Other vessels TVD (mm/mm^2^)	3	1	1	5	3	3	4
Other vessels PVD (mm/mm^2^)	3	1	1	5	3	2	4
Other vessels PPV (%)	16	6.7	5.5	26	18	8.9	23
Other vessels MFI	2.9	0.2	2.6	3.0	3.0	3.0	3.0
All vessels TVD (mm/mm^2^)	18	3.1	10	22	18	17	20
All vessels PVD (mm/mm^2^)	18	3.6	8.3	22	19	17	21
All vessels PPV (%)	96	13	50	100	100	100	100
All vessels MFI	2.9	0.2	2.6	3.0	3.0	2.9	3.0
(C) Velocity/flow							
Small vessel average velocity (*μ*m/sec)	433	140	288	742	407	322	475
Medium vessel average velocity (*μ*m/sec)	441	151	160	670	439	378	534
Large vessel average velocity (*μ*m/sec)	a	a	a	a	a	a	a
All vessel average velocity (*μ*m/sec)	437	126	289	710	412	330	557
Small vessel average flow (*μ*L/sec)	77 000	37 000	16 000	15 0000	76 000	47 000	110 000
Medium vessel average flow (*μ*L/sec)	40 0000	190 000	90 000	740 000	370 000	300 000	500 000
Large vessel average flow (*μ*L/sec)	a	a	a	a	a	a	a
All vessel average flow (*μ*L/sec)	150 000	130 000	16 000	440 000	110 000	47 000	230 000

a Unable to calculate due to small numbers.

There was no evidence in the combined group of significant change over time from 6 h of age until 72 h for any of the videomicroscopy measures (Tables 2CA– and 4CA–). While the change from 6 to 24 h of large vessel length and related parameters did reach significance (mean increase from 0.11 mm, 95th centiles 0.01–0.22, to 0.23 mm, 95th centiles 0.01–0.8, *P* = 0.02), there were few readings with these large vessels present (15 of 42) and so normality of the data could not be guaranteed. Nonparametric testing did not confirm the significance of this change.

When analyzed separately by sex, there were no significant changes over the first 72 h for the male infants for any of the videomicroscopy measures. In the female infants, there was a significant change with time for two parameters only, the measured length of small vessels that were perfused and the derived measure of all vessel PVD, both of which increased marginally but significantly over time.

To investigate the stability over time of the individual measures, partial correlation analysis, with adjustment for sex, was undertaken between the assessment time points. While there were no significant relationships between an individual parameter at 6 h of age and 24 h of age, nor between 24 and 72 h, there was a close relationship between the structural measures of detected vessel length (0.73, *P* = 0.003), small vessel length (0.65, *P* = 0.011), and vessel density (0.73 *P* = 0.003) between the 6‐ and 72‐h time points.

**Figure 2:**
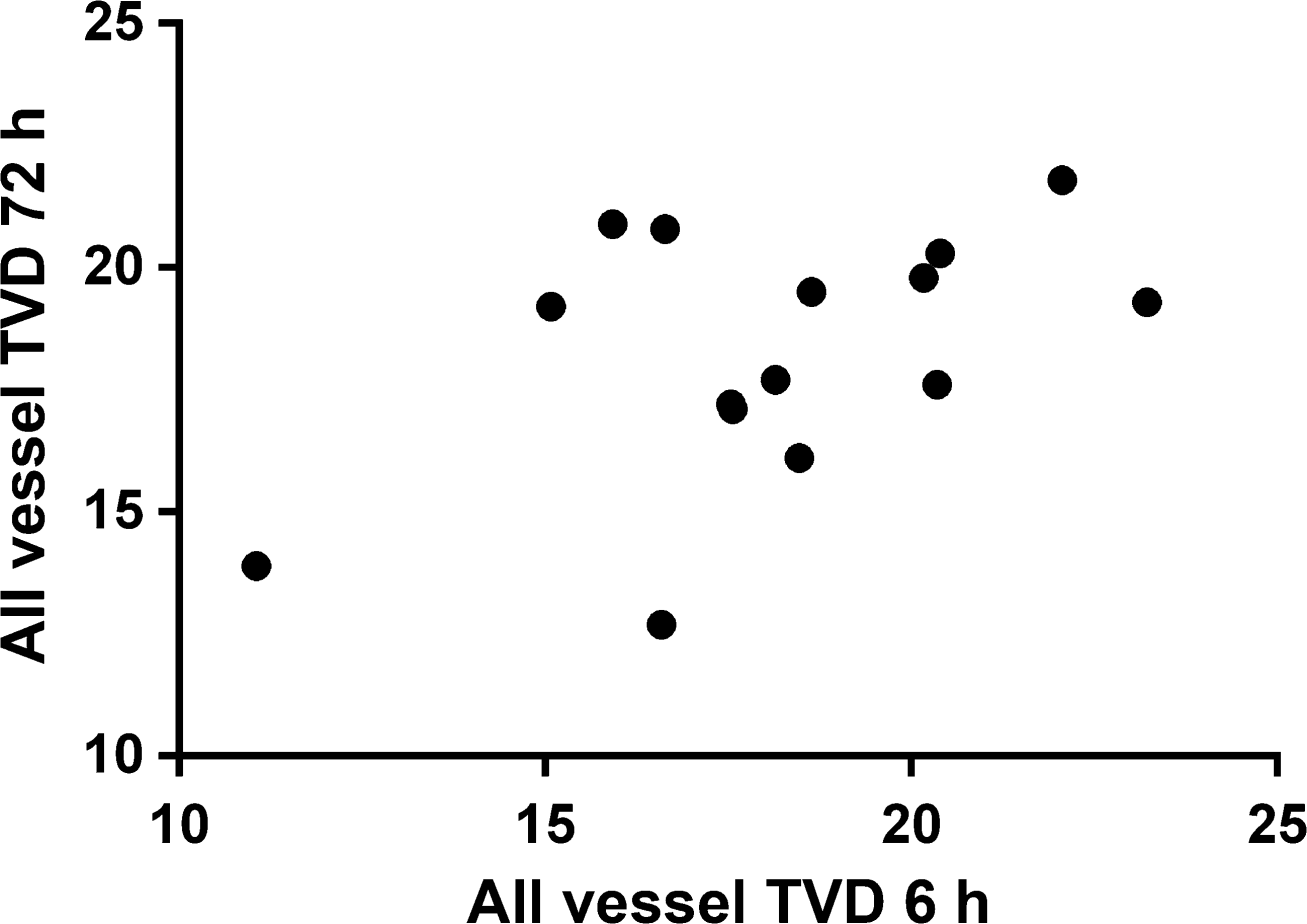
Correlation plot of all vessel TVD (mm/mm^2^) between 6‐ and 72‐h time points. Partial correlation, adjusted for sex, 0.80 (*P* = 0.006).

The same process was undertaken with the semiquantitative functional indices. Of these, there were no relationships between individual measures comparing 6 and 24 h, and 24 and 72 h. Again, there were significant correlations for small vessel TVD (0.64, *P* = 0.048) and all vessel TVD (0.80, *P* = 0.006) between 6‐ and 72‐h values (Fig. 2).

Finally, the quantitative velocity/flow measures were compared over time but showed no significant temporal correlations. There were no significant effects of other maternal parameters, including BMI.

Exclusion of the infants whose mothers received perinatal vasoactive medication did not result in any significant changes to the above observations. Similarly, removing the babies born to smokers, mothers with hypertension or gestational diabetes did not change the results. Exclusion of the two SGA infants in the female analysis meant that the all vessel PVD was no longer significant but the small vessel length remained so. There were too few SGA infants to analyze separately and their results were within the ranges described in the tables.

## Discussion

These observations of the term well newborn provide baseline measures for studies into the use of sidestream dark‐field OPS videomicroscopy in infants from pathological pregnancies or in premature infants. They confirm the practical utility of the ear conch as an appropriate investigation site for microcirculatory videomicroscopy of the newborn.

OPS studies in adults have described parameters in the microvascular circulation of the sublingual and intestinal circulations (De Backer et al. 2010; Sharawy et al. 2011; Ayhan et al. 2013; Donndorf et al. 2014). They have been shown to be useful in assessing systemic shock in the intensive care unit (Wiessner et al. 2009) and also local perfusion changes in surgically created ostomies (Boerma et al. 2007; Turek et al. 2011). Unlike the neonatal skin, the skin of the adult is too thick to reliably view the subcutaneous circulation, but videomicroscopy has been used for both structural and functional assessment of dermal loops for comparisons of functional capillary density (FCD) between different groups (Treu et al. 2011). In comparison with our studies, the values from adult sublingual studies show mainly outcomes based around the semiquantitative indices, especially the MFI. The values for other areas show generally lower velocities and similar or lower functional vessel densities, reflecting the hyperdynamic nature of the neonatal circulation and the restriction of most adult studies to very ill intensive care patients.

OPS studies in older children are few but follow a similar pattern to those of adults, with most being sublingual studies (Top et al. 2011a,b; Nussbaum et al. 2014), with a few from other sites (Kroth et al. 2008). Again, intensive care is the most common clinical assessment, with infection being the commonest clinical entity studied (Weidlich et al. 2009). Few studies have looked at the skin of postneonatal infants, for the same reasons of optical opacity as in adults (Top et al. 2011d). In comparison with our studies, the values from childhood sublingual studies show similar values, although across less parameters with smaller numbers and higher variability due to disease states. For example, Top et al. (2011c) looked at 18 pediatric intensive care patients but only reported on MFI and FCD. They showed that a failure to increase vessel recruitment over treatment days was associated with increased mortality. They went on to demonstrate microcirculatory changes with inhaled vasodilators, but only on eight patients and the one measure, FCD (Top et al. 2011b). Nussbaum et al. (2014) studied 14 diabetic children and controls using similar methodology to our study but only reported MFI, TVD, and vessel coverage (%).

Other human neonatal studies have used videomicroscopy (Genzel‐Boroviczeny et al. 2004; Schwepcke et al. 2013), using different examination sites and a variety of clinical situations, including prematurity (Kroth et al. 2008) and sepsis (Alba‐Alejandre et al. 2013). Kroth et al. (2008) reported 25 significantly preterm infants but only for vessel density and measurements did not commence until near the end of week one of life. Alba‐Alejandre et al. (2013) did report using the same ear conch site in term newborns with or without biochemical evidence of inflammation. In their 31 normal term newborns, the values of continuous flow percentage (IQR: 87–94%) were generally higher than ours (IQR: 73–88% on day 2) but their reported vessel density, defined as De Backer score, was lower (6.2–6.8/mm) compared to ours (11–14/mm). Of note, these reciprocal differences would have resulted in similar total tissue flows and suggest some value in assessing a variety of measures. The reasons for these differences in the limited parameters could relate to infant care practices, hospital environment, or ethnic differences but these would be purely speculative until assessed further. In comparison with our results, the published literature generally reduces the large variety of parameters to semiquantitative indices and the subject numbers studied are too small to investigate the range of normal values within this population. In keeping with our findings, measures of small vessel density appear to be most related to physiological changes (Alba‐Alejandre et al. 2013; Nussbaum et al. 2014). This is a similar finding to a recent review of sidestream dark‐field videomicroscopy that also concluded that the quantitative assessments may be more reliable than some of the indexing methodologies (Petersen et al. 2014).

The most significant recent publication in this area is that of van den Berg et al. (2015). This looks at the reproducibility of microvascular videomicroscopy assessment in a similar population of term neonates. They assessed both buccal (as a proxy for the adult sublingual assessment) and underarm areas. They showed adequate imaging on both areas but greater reproducibility of video analysis with the buccal assessment. Neither study included a second operator to obtain data to truly assess reproducibility of the whole process. We have no data from a second video scorer for the assessment of interobserver variation which could be compared with van den Berg et al. (2015) data, however, our masked assessor showed very high intraobserver reproducibility for assessment of the same images. We differ from their conclusion about the sites; our attempts at buccal imaging were unsuccessful due to the very strong sucking reflex in this age group. Our chosen site of the ear conch has several advantages: it was easier to stabilize without unwrapping the upper arm chosen by the van den Berg group, it shares the same arterial origins as the buccal blood supply, and finally, the skin surface is markedly thinner than the limb skin, allowing similar clarity to that shown in their buccal studies. Our values for TVD and PVD are very similar to those quoted in the van den Berg et al. (2015) paper, if large vessels are excluded, although higher if they are not. The large vessels are significant venules with highly variable flow. Their presence in a field of view was inconsistent in our studies and so had a disproportionate influence on the variability of the data. Finally, van den Berg et al. (2015) studied a smaller number of infants (*n* = 25) at only one time point and have reported on fewer parameters. The quoted lack of association between the buccal results and the limb skin data suggests that carotid microcirculation may have different influences on results than the systemic microcirculation and warrants further investigation of the ear conch site to understand if these differences are mucosal versus skin differences or carotid territory versus noncarotid differences.

OPS videomicroscopy combines a number of different assessments including structural changes, functional semiquantitative indices, and quantitative velocity/flow measures. We found that the functional indices, both because of their semiquantitative nature and their mathematical interrelationships, were often closely correlated with each other (data not shown). Given the massive predominance of one vessel size (small vessels: 1–25 *μ*m) and the lack of intermediate vessel flow states in the generally dynamic neonatal vasculature, we would question the usefulness of many of the parameters derived from the analysis software in the neonatal condition, specifically all the large vessel parameters and many of the medium vessel parameters. In addition, the flow indices showed little variation because of the high flow states that predominated. In adults, it has also been noted that small vessel parameters predominate in reflecting clinical status, but this requires assessment in the sick or premature neonate before this conclusion can be fully extrapolated to the human newborn. It should be noted that while the reporting bins for small vessels are quoted within the software as down to 1 *μ*m, the inability to visualize vessels with no red cell column, the physical limitations of the hardware and software and the fact that the smallest capillaries are 5 *μ*m or more, mean that this lower figure is not actually relevant.

The changes observed are in keeping with previous studies of term infants using other microvascular assessment techniques. Laser Doppler flowmetry studies have demonstrated that changes in vascular function observed in more immature infants are no longer significant at term. Specifically, the sex differences seen in very premature infants become less marked with increased maturity at birth and may not be apparent in term infants (Stark et al. 2008b). This is in keeping with the data shown in this study, with no significant differences at any time point for any parameter between the sexes. On the other hand, previous studies in term and near‐term infants over time, using laser Doppler flowmetry, in the context of hypertension in pregnancy have demonstrated sex differences in the pattern of changes observed postnatally (Stark et al. 2009b). This study also demonstrated a significant difference between males and females in the temporal pattern of these changes, with the healthy female term newborn showing a significant increase in at least one measure of small vessel perfusion over time, suggesting a mildly increased level of vasoconstriction at birth in females as compared to male infants, in keeping with previous reported laser Doppler findings (Stark et al. 2009b).

The large vessels parameters were very variable, both in the presence and number, between recordings. These vessels were largely negatively correlated with the smaller vessel changes (data not shown), which have been recommended as the vessels of most interest (Petersen et al. 2014). In addition, the nature of these large vessels, essentially venules, means they were continuously perfused and contributed little to the variation in functional indices. For these reasons, we would recommend that these vessels be avoided in the field of interest for future neonatal studies, although they may remain of interest in changes in older children, such as those with diabetes (Nussbaum et al. 2014).

This study shows significant correlation for both structural and indices values of vessel density measures between the values obtained at 6 h and those at 72 h suggesting stability which may reflect the underlying structural state of the microcirculation at these time points. Interestingly, this was not maintained when comparing either time point with the corresponding 24‐h data. Previous work, using laser Doppler, has suggested that in the sicker or premature infant, the 24‐h time point is associated with significant vasodilation in those at risk and a relative vasoconstriction of those with less illness (Stark et al. 2008a; Dyson et al. 2014a). Validation of this effect in sicker or premature human populations with OPS videomicroscopy has yet to be shown, but significant functional changes around this time point have been demonstrated in a relevant animal model (Dyson et al. 2014a) which demonstrated the same temporal, gestation‐related, and sex‐related differences observed in human infants. Together these data suggest that studies investigating the in utero programming effect on the microcirculation around birth should focus on 6‐ or 72‐h time points. In view of the potential significant early thermoregulatory differences, we would recommend at least 72 h should be considered a standard time point for these longer term studies, with some suggestion that as late as a week of age may be appropriate to avoid early adaptive cardiovascular changes (Top et al. 2011d).

Debate exists as to whether changes seen over the first few days of life represent structural or functional changes (Wright et al. 2012). Certainly previous studies of the newborn using laser Doppler have been associated with changes in functional mediators of vessel dilatation and perfusion, including endothelin (Stark et al. 2011), nitric oxide, carbon monoxide (Stark et al. 2009a), hydrogen sulphide (Dyson et al. 2014b), and measures of sympathetic drive (Stark et al. 2011). However, a number of other vascular processes should be considered, including angiogenic influences. Subsequent differences in apoptotic vascular remodeling, that is known to occur after birth (Kim et al. 2000), could also lead to some of the changes seen, particularly those proposed between preterm and term infants (Wright et al. 2012). Against this, the close association between the early and late structural values implies a certain structural stability with a functional change occurring at around 1 day of age. We would speculate that it is the later time points that will be more predictive of any future structural adaptation, or programming, seen in those born to more pathological pregnancies.

There are a number of limitations to this study. Although all babies were considered well at the first time point, the mothers included those with a variety of obstetric conditions and treatments. Although we were unable to identify an effect of these maternal conditions on our conclusions, the sample sizes at this level were small and so a type 2 statistical error cannot be excluded. While every effort was undertaken to standardize the conditions of each study, and all babies were normothermic at the time of study, we cannot exclude the influence of thermoregulatory effects on our observations. We were unable to undertake interrater variability studies, due to the limitations of patient handling and out of hours personnel availability, although this would have been ideal to add to our intrarater data. Due to the limitations of access to individual infants, the numbers at both time points varied for each comparison. Use of nonparametric comparisons supports the robustness of our findings but does mean that relationships could be demonstrated, where none were seen here, on larger sample studies in the future (type 2 errors). Finally, we used fixed time points and an investigation site that differs from that used by other researchers in this age group. We cannot, therefore, exclude a difference in behavior of the microcirculation at other times or body sites. We used the currently widespread AVA analysis program, but there are a number of other potential approaches. This methodology is semiautomated but still takes at least 30 min for a trained analyst per recording. The development of fully automated systems may assist with reducing analysis time, but the effect of these changes in analysis method on the parameter values, compared to current data, is unknown in any age group, including in the newborn. Other parameters, not in the AVA software used, appear to also correlate with clinical state, such as fractal analysis and other measures of structural complexity (Cheung et al. 2013). Further studies will be required to elucidate the relevance of these vascular branching and complexity analyses in the well and sick neonate.

In conclusion, we describe for the first time in detail, the values observed for the full range of commonly reported OPS videomicroscopy parameters in the microcirculation of the well term newborn. Changes observed over time and by sex of the infant are consistent with previously described patterns. We recommend that studies for mature neonatal microvascular structure are delayed until after 72 h, but that studies of the physiology of transition include the 24‐h time point.

## Conflict of Interest

None declared.
